# Obinutuzumab induces superior B-cell cytotoxicity to rituximab in rheumatoid arthritis and systemic lupus erythematosus patient samples

**DOI:** 10.1093/rheumatology/kex067

**Published:** 2017-04-11

**Authors:** Venkat Reddy, Christian Klein, David A. Isenberg, Martin J. Glennie, Geraldine Cambridge, Mark S. Cragg, Maria J. Leandro

**Affiliations:** 1 Centre for Rheumatology, University College London, London, UK2 Roche Pharmaceutical Research & Early Development, Roche Innovation Center Zurich, Schlieren, Switzerland3 Academic Unit of Cancer Sciences, University of Southampton, Southampton, UK

**Keywords:** rheumatoid arthritis, systemic lupus erythematosus, B cells, rituximab, obinutuzumab

## Abstract

**Objective.** A proportion of RA and SLE patients treated with standard doses of rituximab (RTX) display inefficient B cell deletion and poor clinical responses that can be augmented by delivering higher doses, indicating that standard-dose RTX is a sub-optimal therapy in these patients. This study aimed to investigate whether better responses could be achieved with mechanistically different anti-CD20 mAbs.

**Methods.** We compared RTX with obinutuzumab (OBZ), a new-generation, glycoengineered type II anti-CD20 mAb, in a series of *in vitro* assays measuring B cell cytotoxicity in RA and SLE patient samples.

**Results.** We found that OBZ was at least 2-fold more efficient than RTX at inducing B-cell cytotoxicity in *in vitro* whole blood assays. Dissecting this difference, we found that RTX elicited more potent complement-dependent cellular cytotoxicity than OBZ. In contrast, OBZ was more effective at evoking Fc gamma receptor-mediated effector mechanisms, including activation of NK cells and neutrophils, probably due to stronger interaction with Fc gamma receptors and the ability of OBZ to remain at the cell surface following CD20 engagement, whereas RTX became internalized. OBZ was also more efficient at inducing direct cell death. This was true for all CD19^+^ B cells as a whole and in naïve (IgD^+^CD27^−^) and switched (IgD^−^CD27^+^) memory B cells specifically, a higher frequency of which is associated with poor clinical response after RTX.

**Conclusion.** Taken together, these data provide a mechanistic basis for resistance to rituximab-induced B-cell depletion, and for considering obinutuzumab as an alternative B-cell depleting agent in RA and SLE.

Rheumatology key messages

- Obinutuzumab induces superior B cell cytotoxicity to rituximab in RA and SLE patient samples.
- B cells from RA and SLE patients internalize rituximab more rapidly than obinutuzumab.
- Obinutuzumab is superior to rituximab at evoking Fc gamma receptor-dependent and -independent effector mechanisms in RA and SLE.

## Introduction

Incomplete B cell depletion following treatment with the anti-CD20 mAb rituximab (RTX) is associated with poor clinical response in both RA [1] and SLE [2], whereas enhanced B cell depletion achieved using additional doses of RTX in RA [3] and prolonged duration of depletion are associated with a better clinical response in SLE [4]. Therefore, these data indicate that achieving complete, durable B cell depletion will improve clinical response in both RA and SLE.

B cell subpopulations may be defined as naïve (IgD^+^CD27^−^), unswitched memory cells (IgD^+^CD27^+^), switched memory cells (IgD^−^CD27^+^) and double negative (DN) cells (IgD^−^CD27^−^). Poor clinical response to RTX in both RA and SLE is associated with a higher number and/or frequency of CD27^+^ memory cells [1, 2, 5] and also with DN B cells in RA [5], suggesting that resistance to depletion of different B cell subpopulations is clinically relevant [6]. Further, a greater frequency of IgD^−^CD27^+^ switched memory cells and DN cells, but not IgD^+^CD27^−^ naïve or IgD^+^CD27^+^ unswitched memory cells, was detectable in peripheral blood of patients 4 weeks after a single low dose of RTX (500 mg), prior to organ transplantation. In contrast, B cell composition in lymph nodes and spleen [7] revealed the presence of IgD^+^CD27^−^ naïve and IgD^+^CD27^+^ unswitched memory cells [8, 9], despite opsonization with RTX [8, 9], which suggests that in lymph nodes depletion by RTX was compromised. Collectively, these findings suggest that RTX depletes naïve cells and IgD^+^CD27^+^ unswitched memory cells more efficiently than IgD^−^CD27^+^ switched memory cells and DN cells, particularly in lymphoid tissues [10].

Anti-CD20 mAbs evoke distinct cytotoxic mechanisms: complement-dependent cellular cytotoxicity (CDC), Fc gamma receptor (FcγR)-mediated depletion through cellular effectors including antibody-dependent cellular cytotoxicity (ADCC) and antibody-dependent cellular phagocytosis, and direct cell death (DCD). Good clinical response to rituximab in both RA [11] and SLE [12] is associated with the higher affinity 158 V polymorphism in CD16a (FcγRIIIa) suggesting that FcγR-mediated mechanisms are important for B cell depletion. Moreover, SLE-associated defects in complement [13], NK cells [14, 15] and neutrophils [16] and acquired defects in phagocytosis [16–18] may impact the efficiency of anti-CD20 mAbs [19].

Anti-CD20 mAbs can be categorized as types I and II. Type I anti-CD20 mAbs, such as RTX, redistribute CD20 into lipid rafts, a property that facilitates clustering and complement activation, but also mAb internalization, which is partly driven by cis-mediated engagement of FcγRIIb [20, 21], reducing surface accessible mAb for engagement with FcγR on effector cells [22] such as NK cells, neutrophils and macrophages [23]. Type II mAbs such as obinutuzumab (OBZ, GA101) do not undergo efficient redistribution, clustering or internalization. Accordingly, in follicular and mantle cell lymphoma high target cell expression of FcγRIIb was shown to be associated with poor clinical response to RTX [20, 24].

Other type I mAbs, ofatumumab and ocrelizumab, have been used in clinical trial settings in RA and/or SLE, respectively [25, 26]. To date, no type II mAb has been used in these diseases. However, OBZ has been used in chronic lymphocytic leukaemia and shown to be more effective than RTX [27]. OBZ has also been glycoengineered with an afucosylated Fc facilitating enhanced affinity for CD16a [28], which is the basis of its superior potency in NK-mediated ADCC [29] and antibody-dependent cellular phagocytosis [30]. Therefore, data on the pre-clinical activity of RTX and OBZ in RA and SLE would be of clear clinical importance to understanding whether OBZ may at least partly overcome autoimmune disease-related resistance mechanisms.

Our previous work showed that internalization of RTX compromised its ability to delete B cells *in vitro* and that glycosylated OBZ was superior to RTX in whole blood B cell depletion assays in both RA and SLE [31]. Here, we compared the ability of RTX and OBZ to evoke different effector mechanisms and delete target B cells from patients with RA and SLE. We show that OBZ is at least 2-fold more efficient than RTX at inducing cytotoxicity of these B cells, that it internalizes less rapidly than RTX from the autoimmune B cells, and that it is less efficient than RTX at recruiting complement, but significantly more potent at evoking FcγR-mediated activation of NK cells and neutrophils as well as FcγR-independent direct cell death. We also show that IgD^−^CD27^+^ switched memory cells and DN cells express significantly lower levels of CD20 than IgD^+^CD27^+^ unswitched memory cells, potentially contributing to their apparent resistance to RTX-induced depletion.

## Methods

All participants of this study provided consent according to the Declaration of Helsinki and this study was approved by the National Research Ethics Service committee, London-Bentham. All patients with RA satisfied the ACR/EULAR classification criteria [32] and all patients with SLE met the ACR classification criteria [33]. The patient demographics are shown in the supplementary Tables S1 and S2, available at *Rheumatology* Online.

### Antibodies and reagents

Anti-CD20 mAbs used in the studies include RTX, OBZ and non-glycoengineered, wild-type glycosylated OBZ (OBZ_Gly_) and in some experiments OBZ with a mutated Fc portion (P329G LALA) that does not engage any Fc-mediated effector functions [34] (OBZ-PG LALA). Roche Innovation Center Zürich, Switzerland generated all anti-CD20 mAbs except RTX, which was a kind gift from the pharmacy of University College Hospital, UK, and AT10 (FcγRII antagonist) [35] was produced in-house.

### Flow cytometry and B cell isolation

Fluorochrome-conjugated mAbs anti-CD3 (phycoerythrin (PE)-Cy7), anti-CD15 (FITC): anti-CD16 (allophycoyanin), anti-CD19 (Alexa Fluor 700), anti-CD45 (PE), anti-CD56 (PE), anti-CD107a (Brilliant Violet 421), anti-CD11b (PE) and anti-CD62L (allophycoyanin), and propidium iodide (PI) and annexin V (Av) (FITC) were obtained from BD Biosciences (Oxford, UK) and Biolegend, London, UK. In addition to forward- and side-scatter characteristics, we identified B cells as CD19^+^, T cells as CD3^+^, NK cells as CD3^−^56^+^ and neutrophils as CD15^+^ by flow cytometry using a Becton Dickinson LSR Fortessa cell analyzer. Peripheral blood mononuclear cells were separated from whole blood by Ficoll-Hypaque density gradient and B cells were isolated using EasySep Human B Cell Enrichment Kit (Stemcell Technologies, Cambridge, UK).

### Whole blood B cell depletion assays

Briefly, 300 μl of freshly drawn whole blood anti-coagulated with heparin was incubated with or without mAbs at 1 μg/ml for 24 h at 37 °C and 5% CO_2_ before analysing with a flow cytometer, as described previously [31]. The percentage B cell depletion was calculated from the proportion of B cells to T cells remaining after treatment and defined as the cytotoxicity index (CTI) as described previously [28, 31].

### Surface fluorescence-quenching assays

Surface fluorescence-quenching assays were performed as described previously [23, 31] to assess internalization of mAbs by B cells. Isolated B cells were incubated for 6 h with Alexa-488 conjugated mAbs at a concentration of 5 μg/ml before analysing by flow cytometry.

### CDC cytotoxicity assays

CDC assays were performed as previously described [36]. Isolated B cells were incubated with mAbs at a concentration of 10 μg/ml for 30 min at 37 °C and 5% CO_2_ stained with anti-CD19, Av and PI and the frequency of CD19^+^Av^+^PI^+^ cells assessed by flow cytometry. We used freshly collected normal healthy human serum as a source of complement and part of the serum was heat inactivated at 56 °C for 30 min to produce heat inactivated serum (HIS). The ability of mAbs to induce CDC was assessed by the relative frequency of CD19^+^Av^+^PI^+^ cells in samples incubated either with normal healthy serum or with HIS.

### Direct cell death

Isolated B cells were incubated in RPMI supplemented with 10% heat inactivated fetal calf serum with or without mAbs at a concentration of 10 μg/ml for 6 h at 37 °C and 5% CO_2_ and stained and analysed as for CDC. The frequency of CD19^+^Av^+^ cells in samples incubated with mAbs compared with samples incubated without mAbs represented the ability of mAbs to induce DCD.

### NK cell degranulation assays

NK cell degranulation was assessed using samples from the whole blood B cell depletion assay by measuring the expression of CD107a or lysosome associated membrane protein 1, which is up-regulated upon activation of NK cells and correlates with NK cell mediated ADCC [37, 38]. The extent of CD16a loss was also used as an indirect measure of NK cell activation [39, 40].

### Neutrophil activation assays

We assessed neutrophil activation in the whole blood assay by measuring increases in the mean fluorescent intensity (MFI) of CD11b or decreases in MFI of CD62L on CD15^+^ neutrophils by flow cytometry [41, 42] in samples incubated with mAbs compared with samples incubated without mAbs.

### Statistical analysis

Data were analysed using Prism version 5.0 (GraphPad Software, La Jolla, CA, USA). The Mann–Whitney test or Wilcoxon’s matched-pairs signed rank test was used to compare groups as appropriate. Spearman’s correlation coefficient was used to analyse for correlation.

## Results

### Type II mAbs are more efficient than type I at inducing B cell cytotoxicity

**Fig. 1:**
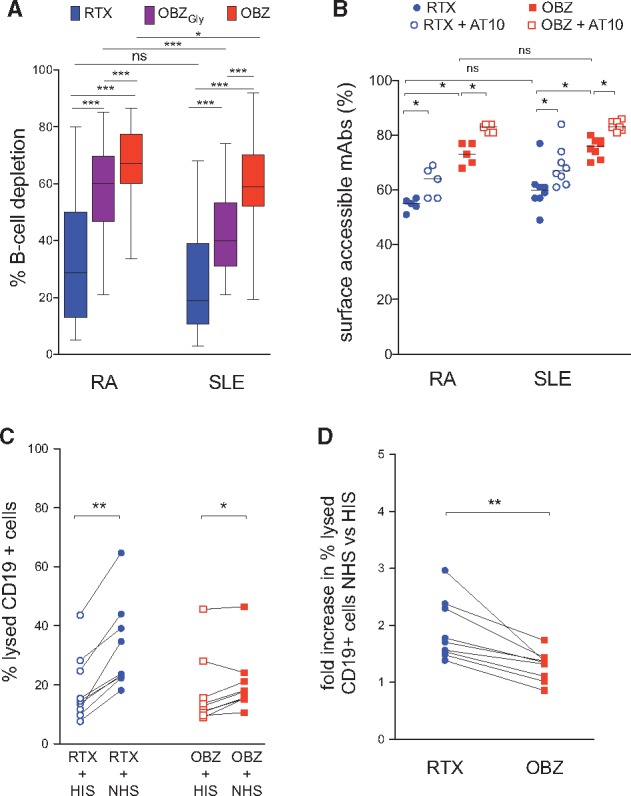
Whole blood B cell depletion, internalization and CDC elicited by anti-CD20 mAbs in RA and SLE patient samples (**A**) Whole blood B-cell depletion in samples from patients with RA (n = 31) and SLE (n = 34). The horizontal line in the box represents the median, the box represents the interquartile range and the whiskers represent the range. (**B**) Surface fluorescence-quenching assay in RA (n = 5) and SLE (n = 8) samples with or without prior incubation with anti-FcγRII blocking mAb, AT10. (**C**) The frequency of lysed CD19^+^Av^+^PI^+^ B cells in SLE (n = 9) samples. (**D**) The fold increase in samples incubated with normal healthy serum (NHS) *vs* heat inactivated serum (HIS). *P < 0.05; **P < 0.005; ***P < 0.0001; and NS, not significant. OBZ_Gly_, obinutuzumab with glycosylated Fc; OBZ, obinutuzumab.

To assess the effect of types I and II mAbs on B cell cytotoxicity in RA and SLE samples, whole blood B cell depletion assays were performed as described previously [31] (supplementary Fig. S1, available at *Rheumatology* Online). OBZ was >2-fold more efficient than RTX at deleting B cells from patients with RA (n = 31) and SLE (n = 34) and both non-glycomodified OBZ_Gly_ and OBZ were more efficient than RTX, in all samples tested (Fig. 1A and supplementary Table S3, available at *Rheumatology* Online). In both RA and SLE, the median CTI of OBZ was significantly greater than the CTI of OBZ_Gly_ and RTX. The CTI of OBZ_Gly_ was significantly higher than the CTI of RTX in both RA and SLE. In RA, the median (interquartile range) CTI of RTX, OBZ_Gly_ and OBZ was 29 (13–50), 60 (47–70) and 67 (60–77), respectively, and in SLE was 19 (11–39), 40 (31–53) and 59 (52–70), respectively. Both type II anti-CD20 mAbs, OBZ_Gly_ and OBZ, demonstrated superior efficiency of B cell cytotoxicity to the type I anti-CD20 mAb, RTX, in all individual samples from patients with RA and SLE (data not shown).

There were no significant correlations between CTI of RTX or CTI of OBZ and patient’s age, serum complement and/or IgA levels in samples from patients with SLE (data not shown).

Thus, in both RA and SLE, there was a hierarchy of mAb-induced B cell depletion: RTX < OBZ_Gly_ < OBZ. The superior efficiency of OBZ_Gly_ (having a non-glycomodified Fc similar to RTX) suggests that its type II nature accounts for the difference between the two types of mAbs in the efficiency of B cell depletion in the whole blood assay, whereas the increased efficiency of OBZ compared with OBZ_Gly_ is attributable to afucosylation of the Fc portion.

### B-cells internalize RTX more rapidly than OBZ

RTX was internalized more extensively than OBZ after 6 h of incubation with a median (range) percentage of surface accessible RTX *vs* OBZ of 55 (51–57) *vs* 83 (81–84), respectively, in RA (n = 5) and 60 (49–77) *vs* 76 (70–80), respectively, in SLE (n = 8) (Fig. 1B). Internalization of RTX and, to a smaller extent, OBZ, was partially inhibited in the presence of the FcγRII-blocking mAb AT10 (Fig. 1B), similar to our previous observations using a non-glycomodified type II antibody variant [31].

### RTX is more efficient than OBZ at inducing CDC cytotoxicity

The frequency of lysed B cells (CD19^+^Av^+^PI^+^) was significantly greater in samples incubated with RTX in the presence of normal healthy serum compared with HIS (supplementary Fig. S2, available at *Rheumatology* Online) with a median (range) difference of 10.9% (8.1–21%) whereas the difference for OBZ was 4.8% (0.9–6.5%) (Fig. 1C). The mean (s.d.) fold increase in lysed cells in samples incubated with normal healthy serum *vs* HIS was 1.9 (0.5) and 1.2 (0.2) for RTX and OBZ, respectively (Fig. 1D). Thus, RTX was superior to OBZ at evoking CDC.

### OBZ is more efficient than RTX at activating NK cells

**Fig. 2:**
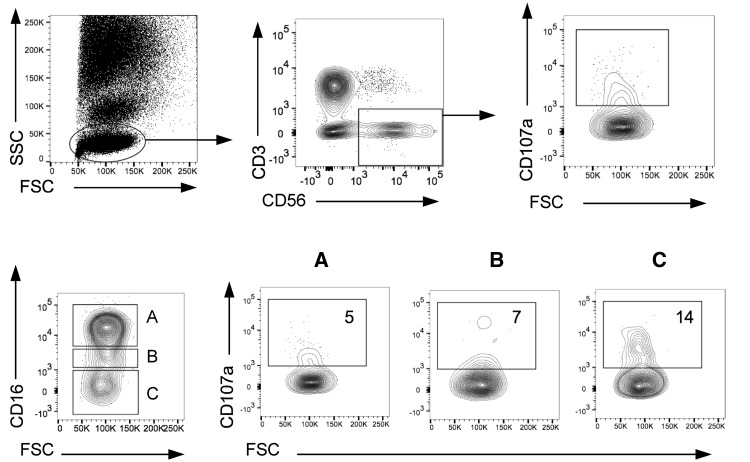
NK cell degranulation assay: relationship between NK cell expression of CD107a and CD16 Flow cytometry-gating strategy to assess NK cell degranulation. Whole blood samples were incubated with or without mAbs for 24 h before analysing by flow cytometry. NK cells were identified based on forward- and side-scatter characteristics and CD56^+^CD3^−^. The frequency of CD3^−^CD56^+^CD107a^+^ cells represented activated/degranulated NK cells. The relative frequency of activated CD107a^+^ NK cells based on CD16 expression in three subpopulations of CD3^−^CD56^+^ NK cells was identified based on the relative expression of CD16 (boxed as **A**, **B** and **C** above). FSC: forward-scatter; SSC: side-scatter.

The ability of the mAbs to induce NK cell activation in the whole blood B cell depletion assay, shown in Fig. 2, allowed assessment of NK cell degranulation (CD107a increase) relative to expression of CD16a. The highest proportion of CD107a^+^ NK (CD3^−^CD56^+^) cells was seen in the CD56^+^CD16^−^ fraction (Fig. 2) suggesting that degranulating NK cells had down-regulated CD16, as previously reported [39].

**Fig. 3:**
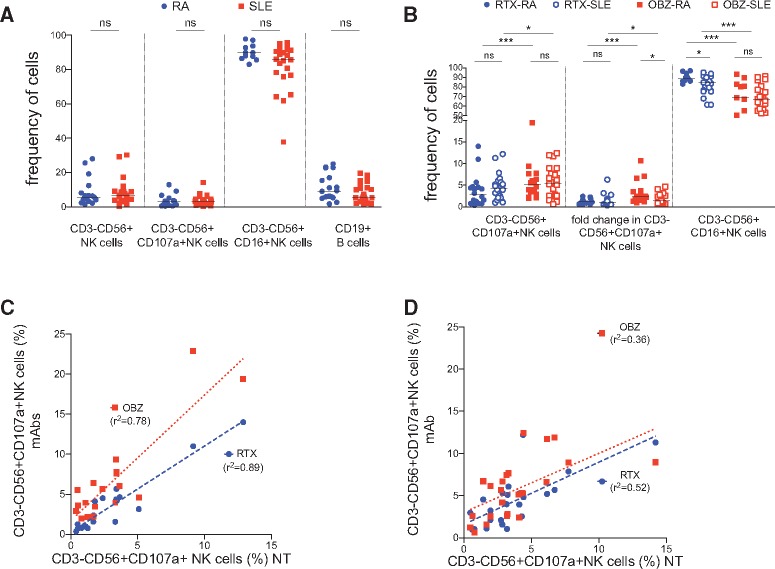
Obinutuzumab is more efficient than rituximab at activating NK cells in RA and SLE patient samples NK cell activation was assessed in whole blood assay using samples from patients with RA (n = 18) and SLE (n = 23) by the frequency of CD3^−^CD56^+^ NK cells, CD3^−^CD56^+^CD107a^+^ NK cells, CD3^−^CD56^−^CD16^+^ NK cells as a percentage of total NK cells and CD19^+^ cells after incubation in the absence (**A**) or presence (**B**) of RTX and OBZ and their relationship in RA (**C**) and SLE (**D**). Horizontal lines represent the median. *P < 0.05; **P < 0.005; ***P < 0.0001; ns: not significant; r^2^: Spearman’s correlation coefficient. NT: not treated; OBZ_Gly_: obinutuzumab with glycosylated Fc; OBZ: obinutuzumab.

In equivalent assays comparing RTX and OBZ, after 24 h of incubation in the absence of mAbs, there was no significant difference in the frequency of NK cells, CD107a^+^ NK cells, CD16^+^ NK cells or B cells between patients with RA (n = 18) and SLE (n = 23) (Fig. 3A). However, in both RA and SLE, the median (range) frequency of CD3^−^CD56^+^CD107a^+^ activated NK cells was significantly higher in samples incubated with OBZ compared with RTX, 5.1% (1.9–22%) *vs* 2.8% (0.3–14%) and 5.5% (0.6–12%) *vs* 4.3% (1.2–8.9%), respectively, and the median (range) frequency of CD16^+^ NK cells was significantly lower, 69% (36–94%) *vs* 89% (83–97%) and 66% (42–91%) *vs* 84% (61–95%), respectively (Fig. 3B). Also, there was a significantly higher fold-increase in the frequency of CD3^−^CD56^+^CD107a^+^ NK cells in samples incubated with OBZ compared with RTX in SLE (Fig. 3B).

NK cell activation, as assessed by either gain of CD107a or loss of CD16, or the fold increase in the frequency of CD3^−^CD56^+^CD107a^+^ NK cells was greater in RA compared with SLE (Fig. 3B). NK cell activation, as assessed by the frequency of CD3^−^CD56^+^CD107a^+^ NK cells, by RTX and OBZ correlated significantly with that in samples incubated without mAbs, with r^2^ = 0.89, P < 0.05 and r^2^ = 0.78, P < 0.05, respectively, in RA (Fig. 3C) and r^2^ = 0.52, P < 0.05 and r^2^ = 0.36, P < 0.05, respectively, in SLE (Fig. 3D). However, correlations were stronger in RA compared with SLE.

**Fig. 4:**
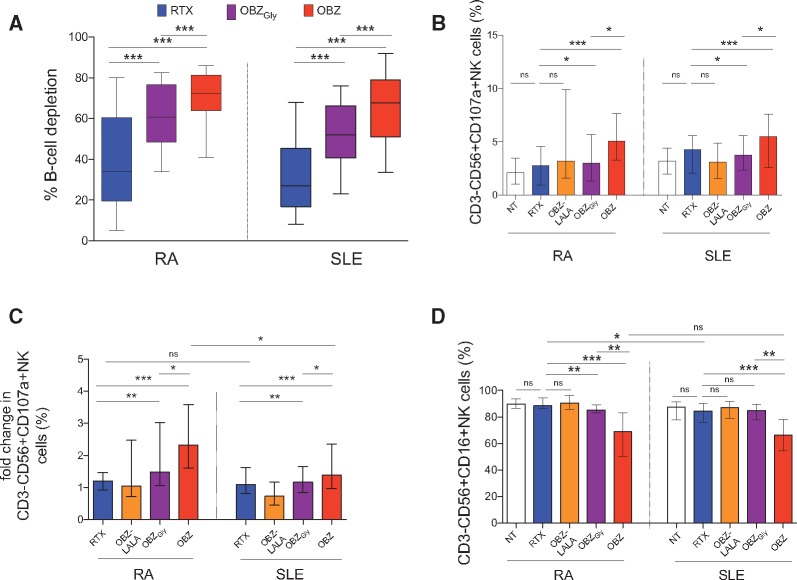
Obinutuzumab induces superior NK cell-mediated cellular cytotoxicity to rituximab in RA and SLE patient samples Whole blood B-cell depletion assays showing (**A**) the percentage B-cell depletion, (**B**) the frequency of CD3^−^CD56^+^CD107a^+^ NK cells, (**C**) the relative increase in % CD3^−^CD56^+^CD107a^+^ NK cells, and (**D**) the frequency of CD3^−^CD56^+^CD16^+^ NK cells in samples from a subgroup of patients with RA (n = 18) and SLE (n = 23) after 24-h incubation with RTX, OBZ-LALA, OBZ_Gly_ and OBZ. For the bar graphs, the error bars represent the median and interquartile ranges. *P < 0.05; **P < 0.005; ***P < 0.0001; ns: not significant. NT: not treated with mAbs; OBZ-LALA: obinutuzumab-PG LALA; OBZ_Gly_: obinutuzumab with glycosylated Fc similar to RTX; OBZ: obinutuzumab.

We next investigated the effect of Fc engineering on activation of NK cells using OBZ_Gly_ and OBZ-PG LALA, which completely lacks FcγR engagement [43]. OBZ was more efficient than OBZ_Gly_ and RTX in depleting B cells in the whole blood assay in both RA (n = 18) and SLE (n = 23) (Fig. 4A) with an increasing hierarchy in the frequency of, and fold-increase in, CD3^−^CD56^+^CD107a^+^ NK cells as follows: no mAbs = OBZ-PG LALA > RTX > OBZ_Gly_ > OBZ (Fig. 4B–D). The frequency of CD3^−^CD56^+^CD16^+^ NK cells was significantly lower in samples incubated with OBZ compared with other samples (Fig. 4D). The frequency of CD3^−^CD56^+^CD16^+^ NK cells was also lower in samples incubated with OBZ_Gly_ compared with RTX in RA, but not SLE (Fig. 4D).

Thus, the ability of mAbs to up-regulate the expression of CD107a on CD3^−^CD56^+^ NK cells was greater in RA compared with SLE, such that the mean fold difference in samples incubated with RTX, OBZ-PG LALA, OBZ_Gly_ and OBZ compared with samples incubated without mAbs was 1.2, 1.5, 1.9 and 3.1, respectively, in RA and 1.5, 0.8, 1.4 and 1.8, respectively, in SLE (Fig. 4C).

### OBZ is more efficient than RTX at activating neutrophils

**Fig. 5:**
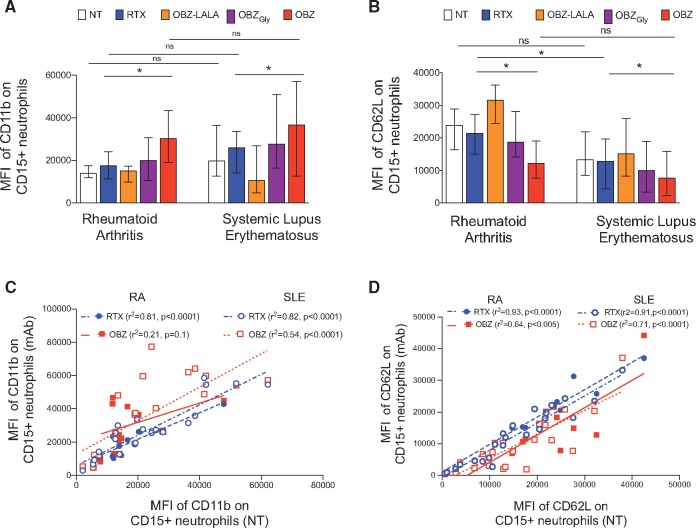
Obinutuzumab is more efficient than rituximab at activating neutrophils in RA and SLE patient samples Whole blood B-cell depletion assays showing (**A**) the mean fluorescence intensity (MFI) of CD11b, (**B**) the MFI of CD62L on CD15^+^ neutrophils, and the relationship between (**C**) the MFI of CD11b and (**D**) the MFI of CD62L on CD15^+^ neutrophils, in samples incubated with or without mAbs in RA (n = 10) and SLE (n = 22) samples. The median and interquartile ranges are represented by the error bars. r^2^: Spearman’s correlation coefficient; *P < 0.05; **P <0.005; ***P < 0.0001; ns: not significant. NT: not treated; OBZ-LALA: obinutuzumab-PG LALA; OBZ_Gly_: obinutuzumab with glycosylated Fc similar to RTX; OBZ: obinutuzumab.

Neutrophils have been proposed as mAb effector cells [41]. We assessed the ability of mAbs to induce neutrophil activation by measuring the expression of CD11b and CD62L, as described previously [42] and shown in supplementary Fig. S3, available at *Rheumatology* Online. CD11b forms part of the β-integrin (Mac-1) complex and genetic variants of this complex have been associated with lupus-related phagocytic defects [44]. Upon neutrophil activation the surface expression of CD11b is up-regulated whereas the expression of the adhesion molecule CD62L is down-regulated [41, 42]. The MFI of CD11b in samples incubated with mAbs was significantly higher in both RA (n = 10) and SLE (n = 22) (Fig. 5A) compared with samples incubated without mAbs. In both RA and SLE, we noted significant correlations between the MFI of CD11b in samples incubated without mAbs and that in samples incubated with RTX (r^2^ = 0.81, 0.82, respectively) whereas significant correlation for OBZ was noted in SLE (r^2^ = 0.81), but not RA (Fig. 5B). We noted a hierarchy in the ability of mAbs to up-regulate CD11b such that the MFI of CD11b was lower in samples incubated with RTX < OBZ_Gly_ < OBZ, as in the case of NK cell activation. The MFI of CD62L was also greater in samples incubated with RTX > OBZ_Gly_ > OBZ (Fig. 5C). In both RA and SLE, we noted significant correlations between the MFI of CD62L in samples incubated without mAbs and that in samples incubated with RTX (r^2^ = 0.93, 0.91, respectively) and OBZ (r^2^ = 0.64, 0.71, respectively) (Fig. 5D). Thus, the hierarchy of mAbs in their ability to activate neutrophils was OBZ > OBZ_Gly_ > RTX. Thus, these data indicated that type II mAbs are superior to RTX in activating neutrophils in the whole blood assay in both RA and SLE samples. OBZ-PG LALA did not elicit significant changes for either marker in both RA (n = 7) and SLE (n = 12) compared with samples incubated without mAbs.

### OBZ is more efficient than RTX at inducing direct cell death

**Fig. 6:**
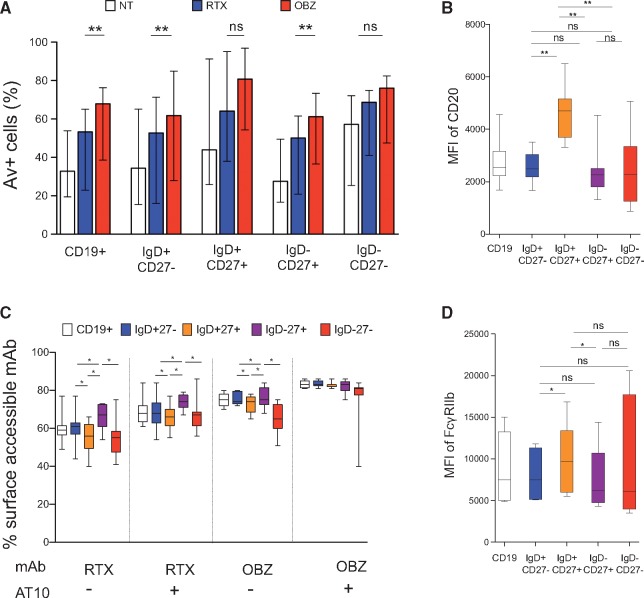
Direct cell death, internalization and expression of CD20 and FcγRIIb in B cell subpopulations In CD19^+^, IgD^+^CD27^−^, IgD^+^CD27^+^, IgD^−^CD27^+^ and IgD^−^CD27^−^ cells from patients with RA (n = 5) and SLE (n = 4), (**A**) the frequency of annexin V^+^ cells in samples, (**B**) the mean fluorescence intensity (MFI) of CD20, (**C**) the frequency of surface accessible mAbs, and (**D**) the MFI of FcγRIIb. The error bars represent the median and interquartile ranges and box and whiskers the interquartile range, and the horizontal line in the box, the median. *P < 0.05; **P < 0.005; ns: not significant. AT10: anti-FcγRII mAb; OBZ: obinutuzumab.

We assessed DCD, using the Av assay as shown in supplementary Fig. S4, available at *Rheumatology* Online. The ability of OBZ to induce DCD was greater than that of RTX for both CD19^+^ cells as a whole and the B cell subpopulations IgD^+^CD27^−^ naïve cells and IgD^−^CD27^+^ switched memory cells (Fig. 6A; RA, n = 5 and SLE, n = 4). The proportion of Av^+^ cells was highest for DN cells > IgD^+^CD27^+^ unswitched memory cells > IgD^−^CD27^+^ switched memory cells > IgD^+^CD27^−^ naïve cells. Nonetheless, OBZ was superior to RTX at inducing DCD.

### Sensitivity of B cell subpopulations to deletion/DCD: relationship with expression of CD20, FcγRIIb and internalization

B cell subpopulations displayed varying ability to internalize mAbs such that IgD^−^CD27^+^ switched memory cells internalized mAbs less than other B cell subpopulations; and IgD^+^CD27^+^ unswitched memory cells internalized mAbs to a greater extent than other B cell subpopulations (Fig. 6C). Antagonizing the effects of FcγRIIb with AT10 significantly reduced internalization in both cases. When compared with naïve and IgD^−^CD27^+^ switched memory cells, IgD^+^CD27^+^ unswitched memory cells had significantly greater expression of CD20 (Fig. 6B) and FcγRIIb (Fig. 6D) and displayed significantly greater ability to internalize mAbs, whereas naïve and IgD^−^CD27^+^ switched memory cells had significantly lower expression of CD20 and FcγRIIb and displayed significantly lower levels of internalization. DN cells had variable levels of expression of CD20 and FcγRIIb, but internalized RTX to a significantly greater extent than IgD^−^CD27^+^ switched memory cells. B cells from both RA and SLE samples consistently displayed low levels of OBZ internalization. Thus, there was no clear relationship between the susceptibility of B cell subpopulations to mAb-induced DCD and the ability to internalize mAbs or to express CD20 or FcγRIIb.

## Discussion

Our data show that OBZ, a type II anti-CD20 mAb with a glycomodified Fc, demonstrated at least 2-fold greater potency at deleting B cells from whole blood samples from patients with RA and SLE compared with RTX. This increased activity of OBZ was effected predominantly through FcγR-mediated effector mechanisms and DCD. In contrast, RTX recruited complement more efficiently for CDC, but was rapidly internalized and significantly less efficient at evoking ADCC and DCD. Our subsequent analysis revealed that the expression of the CD20 target molecule was less on IgD^−^CD27^+^ switched memory and DN cells, perhaps accounting for their relative resistance to removal by RTX.

Target B cells can be deleted with anti-CD20 mAb through multiple mechanisms, with type I mAb engaging complement more effectively than type II mAb. Our findings of superior efficiency of OBZ over RTX at inducing B cell death, despite its inferior ability to recruit complement, are consistent with previous data derived from *in vitro* studies on malignant B cells and/or cell lines [22, 28, 45, 46]. The superior efficiency of OBZ in the whole blood assay was noted in all individual samples. Complement defects are characteristic of certain autoimmune conditions, such as SLE [13], where, we speculate, OBZ may provide a mechanistic advantage over RTX.

The superior efficiency of OBZ in the whole blood assay despite inferior ability to evoke CDC suggests that the predominant mode of action of OBZ is through FcγR-mediated effector mechanisms and/or DCD. While there was no difference between patients with RA and SLE in the frequency of activated NK cells that lacked CD16 expression and/or expressed CD107a, NK cells from patients with both RA and SLE responded less well to stimulation with RTX compared with OBZ. We found that activation of NK cells by anti-CD20 mAbs is also associated with down-regulation of CD16 revealing remarkable differences in activation of NK-cell subpopulations based on the relative expression of CD16 and up-regulation of the degranulation marker, CD107a. Whereas RTX was less efficient at activating NK cells in both RA and SLE, OBZ induced a greater fold-increase in activating NK cells in samples from patients with RA compared with SLE, suggesting SLE-associated NK cell defects may also contribute to poor depletion with RTX [14, 15, 47].

The relative inefficiency of RTX at evoking ADCC *in vitro* may, at least partly, be due to internalization of mAbs leading to reduced engagement with FcγR-bearing effector cells, as shown previously [22, 36]. Afucosylation of Fc increases the affinity of IgG1 mAbs for CD16a with little effect on complement binding [48], which may explain the superior efficiency of OBZ at activating NK cells in the whole blood assay even in the presence of complement [49]. Therefore, the superior efficiency of OBZ at activating NK cells may be attributable to a greater surface accessibility owing to its type II nature and a greater affinity for CD16 conferred by afucosylation of Fc.

Our findings of superior neutrophil activation by OBZ compared with RTX in RA and SLE samples are consistent with studies in malignant B cells [41]. Polymorphisms of CD16b may at least partially account for the variability in mAb-induced activation, most notable for RTX whereas afucosylation may have reduced this variability, as described previously [41]. A number of polymorphisms of CD11b associated with SLE may have contributed to the variability between patients in neutrophil activation [44], but regardless, glycoengineered OBZ was more efficient than wild type OBZ and RTX at inducing neutrophil activation.

Following RTX treatment, a small number of IgD^−^CD27^+^ switched memory cells and DN cells are detectable in the peripheral blood of patients with RA and SLE [5, 6, 50] suggesting relative resistance to depletion by RTX, perhaps due to lower levels of CD20 on IgD^−^CD27^+^ switched memory cells and DN cells compared with IgD^+^CD27^+^ unswitched memory cells. Surface expression of IgD and the activation state of B cells may also influence internalization of mAbs, compromising their cytotoxicity [31]. Regardless, OBZ induced greater DCD *in vitro* in CD19^+^ cells and IgD^−^CD27^+^ switched memory cells from patients with RA and SLE, compared with RTX. These findings are similar to that in malignant B cells [28].

The main limitations of this study are that all experiments were performed *in vitro*. Therefore, these results showing superior efficiency of OBZ to RTX may not translate into more efficient B cell depletion *in vivo* and/or in different tissues such as the lymph node, kidney and joint. Further, concomitant therapies may influence the pharmacokinetics of OBZ and have an impact on its overall efficiency to deplete B cells in patients with RA and SLE.

Disease- and host-associated immune deficiencies may contribute to incomplete depletion with RTX in some patients with RA and SLE leading to worse clinical responses. Phase II clinical studies are on-going to evaluate the efficacy of OBZ in patients with lupus nephritis (NCT02550652) and hypersensitized patients with end-stage renal disease awaiting transplantation (NCT02586051). Our results showing superior efficiency of OBZ over RTX, noted in the whole blood assay, are likely due to FcγR-mediated effector mechanisms and DCD.

This study provides compelling mechanistic reasons for expecting better outcomes with OBZ as an alternative B cell depleting agent for patients with RA, and SLE in particular.

## Supplementary Material

Supplementary Data
