# Supplementary material for: Obinutuzumab induces superior B-cell cytotoxicity to rituximab in rheumatoid arthritis and systemic lupus erythematosus patient samples
Source: Rheumatology (Oxford). 2017 Apr 11;56(7):1227–37. doi: 10.1093/rheumatology/kex067 (PMC5808665; doi:10.1093/rheumatology/kex067)
Supplement: Supplementary Data [file kex067_supp.docx]

**Supplementary data**

**Supplementary Figure S1. Gating strategy for whole blood B-cell depletion assay**

- Lymphocytes were identified by forward- and side-scatter characteristics on flow cytometry and CD45 positivity. B cells were identified as CD19+ cells and T cells as CD3+. The frequency of B cells in samples incubated without anti-CD20 monoclonal antibodies (mAbs) and in samples incubated with rituximab and obinutuzumab are shown.
- **Supplementary Figure S2. Gating strategy for complement-dependent cytotoxicity assay**
- CDC by RTX and OBZ. Isolated B cells were incubated with mAbs either with NHS or HIS for 30 minutes at room temperature before analysing by flow cytometry. The frequency of An V+ PI+ cells represented cell death. HIS: heat inactivated serum; NHS: normal healthy serum; RTX: rituximab; OBZ: Obinutuzumab; An V: Annexin V; PI: propidium iodide.
- **Supplementary Figure S3. Flow cytometry-gating strategy to assess neutrophil activation** After 24-hour incubation, whole blood samples were analysed by flow cytometry. Neutrophils were identified by forward- and side-scatter and CD15 positivity. The mean fluorescence intensity of CD11b and CD62L was analysed on gated neutrophils positive for CD15. FSC: forward-scatter; SSC: side-scatter; NT: not treated with monoclonal antibodies; RTX: rituximab; OBZ-LALA: Obinutuzumab-PG-LALA; OBZ-WT: Obinutuzumab with glycosylated Fc similar to RTX (OBZ_Gly_); OBZ: Obinutuzumab.

**Supplementary Figure S4. Flow cytometry-gating strategy to assess direct cell death**

After 6 hours of incubation without mAbs at 37°c and 5% CO_2_, isolated B-cells were analyzed by flow cytometry. CD19+ B-cells were categorized into naïve (IgD+CD27-), unswitched memory cells (IgD+CD27+), switched memory cells (IgD-CD27+) and double negative cells (IgD-CD27-). The frequency of Annexin V + cells represented direct cell death.

**Supplementary Table S1. Baseline characteristics of patients with Rheumatoid Arthritis**

| patient number | Disease activity DAS-28-ESR | Medications |
| --- | --- | --- |
| 1 | 5.68 | CS, MTX, SSZ |
| 2 | 8.39 | SSZ, HCQ |
| 3 | 5.4 | HCQ, MTX, SSZ |
| 4 | 5.9 | IFX |
| 5 | 3.6 | MTX, SSZ |
| 6 | 2.72 | CS, HCQ |
| 7 | 5.12 | CS, HCQ |
| 8 | 5.7 | SSZ, MTX, Humira |
| 9 | 4.2 | MTX, Humira |
| 10 | 3.8 | IFX |
| 11 | 7.97 | MTX, SSZ |
| 12 | 4.3 | HCQ, Humira |
| 13 | 3.8 | IFX |
| 14 | 5.12 | MTX |
| 15 | 5.4 | MTX, Golimumab |
| 16 | 4.3 | Enbrel |
| 17 | 5.07 | IFX |
| 18 | 3.1 | MTX |
| 19 | 5.5 | MTX, SSZ, Humira |
| 20 | 1.9 | MTX, SSZ, Enbrel |
| 21 | 5.5 | CS, HCQ, MTX |
| 22 | 5.4 | CS |
| 23 | 5.4 | CS, HCQ, LFN |
| 24 | 2.45 | MTX |
| 25 | 2.72 | RTX |
| 26 | 7.4 | MTX, SSZ |
| 27 | 3.8 | CS, AZA |
| 28 | 5.2 | CS |
| 29 | 5.6 | HCQ, MTX, SSZ |
| 30 | 4.17 | CS, MTX |
| 31 | 3.22 | HCQ, MTX, SSZ |

DAS-28-ESR: disease activity score-28- erythrocyte sedimentation rate; LFN: leflunomide; IFX: infliximab; Humira: anti-TNF agent; Enbrel: anti-TNF agent; Golimumab: anti-TNF agent.

**Supplementary Table S2. Baseline characteristics of patients with SLE**

| Patient number | Clinical  manifestations | C3 | Disease activity | BILAG score | Medications |
| --- | --- | --- | --- | --- | --- |
| 1 | LN | 0.73 | active | 21 | CS, HCQ, MMF |
| 2 | non-renal | 1.31 | active | 13 | CS |
| 3 | non-renal | 0.87 | inactive | 0 | CS, HCQ |
| 4 | non-renal | 1.22 | active | 9 | CS, HCQ |
| 5 | non-renal | 1.44 | active | 8 | CS, HCQ |
| 6 | non-renal | 0.94 | inactive | 2 | HCQ |
| 7 | non-renal | 1.13 | inactive | 0 | HCQ |
| 8 | LN | 0.82 | active | 8 | CS, MMF |
| 9 | non-renal | 1.04 | active | 13 | CS, AZA, HCQ |
| 10 | non-renal | 1.17 | active | 10 | CS, AZA |
| 11 | non-renal | 0.82 | inactive | 1 | nil |
| 12 | LN | 0.82 | active | 13 | CS |
| 13 | LN | 1.01 | inactive | 2 | CS, HCQ |
| 14 | non-renal | 1.07 | inactive | 0 | HCQ, MMF |
| 15 | LN | 1.1 | inactive | 1 | CS, HCQ |
| 16 | LN | 0.82 | inactive | 3 | HCQ, MMF |
| 17 | non-renal | 1.01 | inactive | 2 | HCQ |
| 18 | LN | 1.1 | inactive | 0 | HCQ, MTX |
| 19 | non-renal | 0.97 | active | 13 | CS, AZA |
| 20 | non-renal | 1.08 | inactive | 0 | nil |
| 21 | LN | 1.07 | inactive | 1 | CS, HCQ |
| 22 | LN | 0.9 | inactive | 1 | CS, HCQ, MMF |
| 23 | LN | 0.86 | active | 8 | HCQ |
| 24 | non-renal | 1.12 | active | 9 | CS |
| 25 | LN | 0.91 | active | 17 | HCQ |
| 26 | LN | 1.05 | active | 8 | CS |
| 27 | LN | 0.68 | inactive | 2 | CS, AZA, HCQ |
| 28 | LN | 0.67 | inactive | 2 | CS, HCQ |
| 29 | non-renal | 1.04 | active | 9 | HCQ |
| 30 | non-renal | 1.2 | inactive | 1 | CS, HCQ, MMF |
| 31 | non-renal | 0.82 | inactive | 3 | HCQ, AZA |
| 32 | non-renal | 0.7 | inactive | 2 | CS, HCQ, MMF |
| 33 | LN | 1.53 | inactive | 0 | CS |
| 34 | LN | 1.27 | active | 13 | CS, AZA |
| 35 | LN | 1.3 | active | 9 | CS |
| 36 | LN | 0.91 | inactive | 2 | CS, HCQ, MMF |
| 37 | non-renal | 1.42 | inactive | 0 | nil |
| 38 | LN | 0.6 | inactive | 0 | HCQ |
| 39 | LN | 1.1 | inactive | 0 | MTX |
| 40 | non-renal | 1.42 | active | 13 | CS, AZA |
| 41 | LN | 0.73 | active | 10 | CS, HCQ, MMF |
| 42 | LN | 1.05 | inactive | 0 | CS, MMF |
| 43 | non-renal | 1.17 | inactive | 0 | HCQ |

**Table 3. Flow cytometry data from the whole blood B-cell depletion assay**

| patient number | Rheumatoid arthritis | | Systemic lupus erythematosus | |
| --- | --- | --- | --- | --- |
|  | RTX-CTI | OBZ-CTI | RTX-CTI | OBZ-CTI |
| 1 | 13 | 77 | 8 | 58 |
| 2 | 9 | 53 | 41 | 77 |
| 3 | 17 | 40 | 21 | 53 |
| 4 | 12 | 37 | 11 | 49 |
| 5 | 11 | 76 | 18 | 58 |
| 6 | 11 | 64 | 3 | 48 |
| 7 | 13 | 64 | 26 | 68 |
| 8 | 5 | 41 | 6 | 61 |
| 9 | 29 | 77 | 54 | 71 |
| 10 | 35 | 64 | 67 | 70 |
| 11 | 59 | 77 | 15 | 47 |
| 12 | 39 | 75 | 37 | 63 |
| 13 | 18 | 67 | 24 | 53 |
| 14 | 13 | 66 | 39 | 57 |
| 15 | 34 | 63 | 10 | 59 |
| 16 | 18 | 50 | 39 | 47 |
| 17 | 34 | 85 | 17 | 64 |
| 18 | 15 | 76 | 14 | 55 |
| 19 | 50 | 77 | 62 | 82 |
| 20 | 30 | 87 | 10 | 19 |
| 21 | 25 | 75 | 4 | 57 |
| 22 | 68 | 82 | 49 | 79 |
| 23 | 55 | 60 | 32 | 61 |
| 24 | 28 | 65 | 3 | 74 |
| 25 | 56 | 66 | 39 | 80 |
| 26 | 70 | 81 | 9 | 53 |
| 27 | 44 | 86 | 19 | 69 |
| 28 | 67 | 34 | 16 | 43 |
| 29 | 13 | 57 | 28 | 51 |
| 30 | 80 | 85 | 17 | 27 |
| 31 | 34 | 82 | 68 | 79 |
| 32 |  |  | 14 | 64 |
| 33 |  |  | 29 | 59 |
| 34 |  |  | 19 | 92 |

RTX: rituximab; OBZ: obinutuzumab; CTI: cytotoxicity index.
